# Single-Fraction Adjuvant Electronic Brachytherapy after Resection of Conjunctival Carcinoma

**DOI:** 10.3390/cancers13030454

**Published:** 2021-01-26

**Authors:** Gustavo R. Sarria, Solon Serpa, Mario Buitrago, Paola Fuentes Rivera, Diego Ramirez, Frank A. Giordano, Gustavo J. Sarria

**Affiliations:** 1 Department of Radiation Oncology, University Hospital Bonn, University of Bonn, 53127 Bonn, Germany; frank.giordano@ukbonn.de; 2 Department of Ophtalmic Oncology, Instituto Nacional de Enfermedades Neoplasicas, Lima 15038, Peru; sserpa@inen.sld.pe (S.S.); mbuitrago@inen.sld.pe (M.B.); diego_ram2903@hotmail.com (D.R.); 3 Department of Radiotherapy, Instituto Nacional de Enfermedades Neoplasicas, Lima 15038, Peru; pfuentes@inen.sld.pe (P.F.R.); gsarria@inen.sld.pe (G.J.S.)

**Keywords:** conjunctival carcinoma, electronic brachytherapy, low-energy X-rays, kilovoltage, single-fraction brachytherapy

## Abstract

**Simple Summary:**

A centralized distribution of specialized oncologic facilities is a widely repeated situation in many latitudes around the globe, limiting the patient’s access options to specialized treatments. Strategies to alleviate the overpassed attention capacities in low- and middle-income countries, such as Peru, have driven the attention of practitioners towards hypofractionated treatments. In order to shorten treatment times and hospital visits, treating ocular conjunctival carcinoma with a single-fraction electronic brachytherapy approach arises as a novel option, which further increases the current therapeutic arsenal against this entity. We aim to report the clinical findings of this treatment modality, in terms of feasibility, oncological outcomes and toxicity profile, while opening a new possibility of diminishing patient- and health care-related financial impact.

**Abstract:**

A retrospective study was performed to assess the outcomes of a single-fraction adjuvant electronic brachytherapy (e-BT) approach for patients with squamous cell conjunctival carcinoma (SCCC). Forty-seven patients with T1–T3 SCCC were included. All patients underwent surgery followed by a single-fraction adjuvant e-BT with a porTable 50-kV device. Depending on margins, e-BT doses ranged between 18 to 22 Gy prescribed at 2 mm depth, resembling equivalent doses in 2 Gy (EQD2) per fraction of 46–66 Gy (α/β ratio of 8–10 Gy and a relative biological effect (RBE) of 1.3). The median age was 69 (29–87) years. Most tumors were T1 (40.4%) or T2 (57.5%) with a median size of 7 mm (1.5–20). Margins were positive in 40.4% of cases. The median time from surgery to e-BT was nine weeks (0–37). After a median follow-up of 24 (17–40) months, recurrence occurred in only two patients (6 and 7 months after e-BT), yielding a median disease-free survival (DFS) of 24 (6–40) months and DFS at two years of 95.7%. Acute grade 2 conjunctivitis occurred in 25.5%. E-BT is a safe and effective for SCCC treatment, with clinical and logistic advantages compared to classical methods. Longer follow-up and prospective assessment are warranted.

## 1. Introduction

Squamous cell conjunctival carcinoma (SCCC) represents the most frequent malignancy of the ocular surface [1]. Incidences are higher in both elderly population and tropical areas, as most of the reported literature stems from countries where patients are more frequently exposed to high ultra violet radiation levels [2,3,4]. Additional related factors are human papilloma virus (HPV) and human immunodeficiency virus (HIV) infections [5,6]. SCCC constitutes a neoplasm of particular low regional and distant metastasis risk, and thus cancer-related morbidity remains low [7,8]. Logically, the key goal of cancer care for patients with excellent prognosis is to keep toxicity as low as possible, which in SCCC management is challenging due to the inherent location of the tumor [9,10]. The cornerstone treatment in the upfront management of these tumors is surgery [2,11]. Various reports suggest a minimum safety resection margin of 3–4 mm, which in some cases could not be achievable due to foreseen impaired functional outcomes [12,13,14]. Moreover, surgery alone is still potentially related to high recurrence rates (0–56%) [10,15,16,17] and different adjuvant treatment options, namely topical chemotherapy, external beam radiotherapy (EBRT) or brachytherapy might be necessary to reduce local failure rates [12,16,18,19,20].

We report herein the outcomes of patients who received adjuvant electronic brachytherapy (e-BT), representing a low-cost alternative to standard brachytherapy or EBRT. E-BT is carried out using a miniaturized source that emits low-energy X-rays (max. 50 kV), which, due to their unique physical characteristics, are absorbed rapidly and thus establish a steep dose gradient [21,22]. In contrast to EBRT, e-BT allows the high-dose single-fraction treatments, which eliminates extended (fractionated) dose delivery.

## 2. Results

### 2.1. Patient’s Features

Forty-seven patients who underwent surgical resection were included in the analysis. The median age was 69 years (range: 29–87 years), with 55.3% male and 44.7% female patients. Place of residence/origin was Lima for 51.1% of the patients and 48.9% for other cities. Primary surgery was performed at Instituto Nacional de Enfermedades Neoplasicas in 55.4%. All patients from the two participating institutions underwent the same procedure consisting of wide local excision, whilst 12.8% had re-excision due to initial positive margins and according to further procedural feasibility. Final margin status was R0 in 55.4% and R1 (peripheral) in 40.4%; one patient had no available information regarding this parameter. The median time from surgery to e-BT was nine weeks (0–37). Tumor stages were T1 in 40.4%, T2 in 57.5%, and T3 in 2.1% (*n* = 1). The median tumor size was 7 mm (1.5–20) (largest diameter). Schemes of 18 Gy (55.4%), 20 Gy (19.1%) and 22 Gy (25.5%) were prescribed in correlation to the margin status, at 2 mm depth in every case. The only patient with an undefined margins status received 22 Gy as well. The 1-cm and 2-cm diameter flat applicators were used in 91.5% and 8.5%, respectively.

### 2.2. Treatment Outcomes

**Figure 1:**
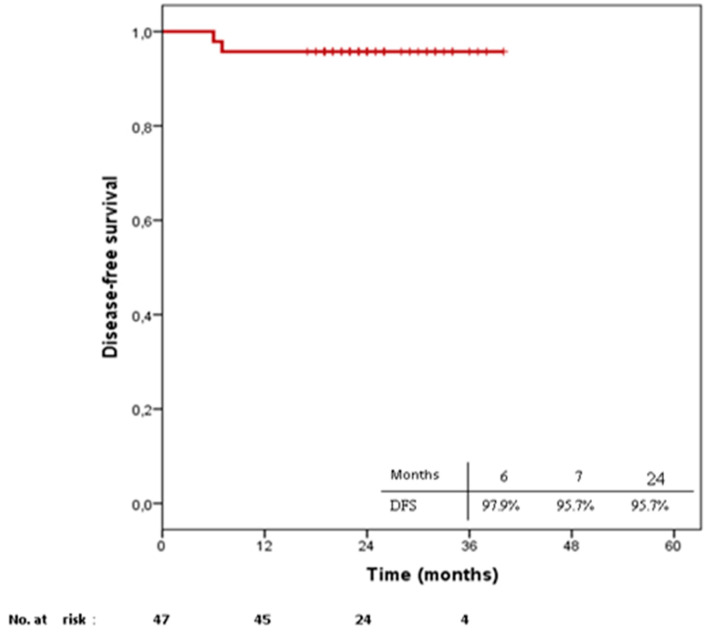
Estimated disease-free survival curves according to the Kaplan–Meier method.

After a median follow-up of 24 months (17–40), median disease-free survival (DFS) was 24 months (6–40) with two patients developing local recurrence after six and seven months post e-BT, respectively. Of these patients, one had T1 disease with R0 margins and one T2 with R1. The estimated 2-year DFS remained at 95.7%. No nodal or distant metastases were reported during follow-up (Figure 1).

Acute G2 conjunctivitis was reported in 25.5% (*n* = 12) of the cohort, with no further acute, late, or higher grade events. All cases clinically improved with topic steroids between one to three months after e-BT. No other secondary events, such as dry eye, cataracts, or ulcers were evidenced during the follow-up period. No significant statistical relationship was found comparing both non-toxicity (*n* = 35) and toxicity (*n* = 12) groups with toxicity incidence and age, tumor size, weeks until application, or applicator size (Table 1). No assessment regarding T stage and doses in relationship to toxicity incidence was feasible due to sample size. Exemplary dose distribution on organs at risk (OARs) from a retrospective simulation is displayed in Table 2.

**Figure 2:**
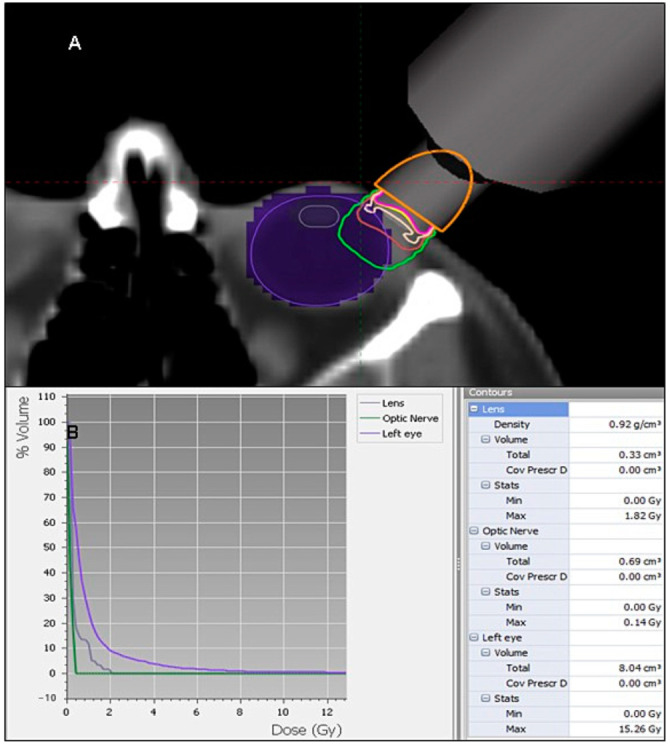
Exemplary treatment simulation and dose distribution of a 1-cm applicator. (**A**) The beige, brown, and green curves represent the 100%, 50%, and 20% isodose lines, respectively; (**B**) dose–volume histogram and D_max_ on OARs.

The sharp fall-off gradient of electronic brachytherapy allows protecting extra- und intraocular healthy structures. Figure 2 shows an exemplary retrospective simulation performed on a randomly selected CT.

## 3. Discussion

To our knowledge, we are the first group worldwide to report the outcomes of e-BT in this scenario. In addition to a previous publication regarding treatment safety and toxicity profile [23], these current results highlight the value of this approach, in matters of toxicity, disease control, and logistic advantage. The latter feature yields a special benefit, as high influx facilities struggle permanently in matters of extended waiting lists for EBRT slots [24]. The versatility of this device could be as well regarded as a potential logistic advantage, as different entities and anatomical regions could be as well treated with it [25,26,27,28].

Probably, due to the low incidence of this malignancy, its standard management is still to be defined [29]. Despite the relationship of this entity to low regional- or distant-failure rates when promptly treated [7,8], the local failure rates after a pure surgical approach could remain elevated, according to various publications [14,30,31]. Most of the surgical series recommend an excision margin of 3–4 mm; however, this might not be reachable in some cases due to foreseen impaired functional outcome [12,15]. Furthermore, even in cases where adequate margins were achieved, the local failure rates remained to be unacceptably high, deriving in additional adjuvant treatment [10]. Different approaches such as topical chemotherapeutic agents (5-fluorouracil [5-FU], Mitomycin C [MMC]), or cryotherapy have also proven to be beneficial [32,33,34,35]; however, the required average chemotherapy time of four weeks (cycles) might be a limiting factor for treatment adherence and acquisition (due to irregular drug availability and costs) [36]. With a varying range of prices between 37–75 USD per 1–2 cycles, this would result in being unaffordable for many patients in developing countries if not covered by an insurance [19,37,38], in comparison to an average 90 USD e-BT cost entirely assumed by the Peruvian public health system. Toxicity rates might represent an additional fact for treatment declining, as the rate of overall adverse events and in particular conjunctivitis of this strategy reaches as high as 48–69%, which could greatly impair the patient’s life quality [29,35,39,40,41].

Historical local control reports show recurrence rates within the range of 0–56%, after a sole-surgery approach and depending on the margins status [10,15,16,17]. Adding topical adjuvant chemotherapy improves the local control rates, as two previous publications (*n* = 196) comparing surgery alone with combined therapy have confirmed this, with reported local failures (LF) of 3–11% after one year but up to 25% after five years [16,29,36]. It is important to remark that most of the recurrences for this entity tend to occur during the first year of treatment [1], which is consistent with our findings. However, long-term follow-up should be as well performed, as the risk of later failure might be considerable [10].

A dosimetric advantage of kilovoltage application on this kind of tissue surface is also remarkable, due to its sharp fall-off dose profile compared to photon- or electron-based teletherapy units [21,22]. This feature allows better OAR sparing, as historical data report elevated rates of cataract, higher-grade conjunctivitis, uveitis and ulcer, amongst others [42,43,44]. Newly published data suggested that proton therapy might also be an optimal EBRT option. Outcomes from a small sample cohort, including 35 patients treated with this modality, described a 12% rate of local failure after two years [45]. However, the well-known elevated costs and scarce availability of proton therapy centers across the globe still undermine this possibility. Brachytherapy plaques have also demonstrated their value in terms of disease control; however, gathered data to the moment seem to show greater rates of adverse events compared to this approach (~25%) [20,46]. Additionally, the dosimetric characteristics of isotope plaques require more strict radiation protection measures which might raise the logistics costs *per se* and, moreover, depending on each individual plaque cost [47,48]. Furthermore, the need of hospitalization for some plaques and several-days permanence could represent, as well, a mostly limiting logistic factor in developing countries [20]. The particular features of Strontium-90 (Sr^90^) result in being interesting in matters of comparison due to the low-penetration profile and 10.2 Gy/h output rate of this β-emitter, yielding outstanding surface coverage [49]. An isodose modeling performed by Barbosa et al. describing dose delivery per hour could allow estimating absorbed doses per each OAR. For example, doses of 1.08 Gy, 0.77 Gy, and 2.36 Gy could reach the lenses, retina, and optic nerve after 1 h exposure to the source, respectively [50]. However, this may vary according to the position of the plaque and desired dose prescription. Clinical data on SCCC treatment with Sr^90^ have been addressed in the past. A classical report revealed excellent control rates with three local failures out of 123 patients who underwent this 30 Gy single-fraction treatment, with negligible adverse events incidence [51]. It is worth mentioning the estimated time equivalence of this approach, requiring delivery times of approximately 1 to 28 min, depending on isotope decay, fractionation, and dose and depth prescription, in comparison to the estimated single 5–7 min. duration with our technique [46,51].

The rationale for the G2 toxicity classification in this cohort is the use of topical corticosteroids, as, per the Common Terminology Criteria for Adverse Events (CTCAE) v 4.03 [52], the sole usage of topical treatment is enough for raising the grading [52]. Despite this, most of the patients remained asymptomatic or were mildly symptomatic and underwent treatment due to local protocol, whilst no further events were found during follow-up. This toxicity profile resembles previous findings from a series of fractionated stereotactic radiotherapy (SBRT) delivery, suggesting that dose fractionation might not be highly relevant in order to decrease toxicity [53,54,55]. No statistical association was found between the analyzed features and the toxicity incidence, most likely because of the sample size. Nevertheless, we consider that attention should be focused on particular features, like treatment field size (2 cm diameter applicators), doses, and prescription depth, which in different settings than the ones used for this study might be potentially related to major toxicity events.

Despite the retrospective nature of this report, these data are quite consistent regarding patient selection, homogeneity of treatment, and follow-up protocols; however, the small sample size must be noted considering the overall low incidence and socio-demographic distribution of this entity, which might lead to underpowered statistical values and recruiting and long-term follow-up restrictions. With regard to the latter, the patients´ place of residence poses a limitation for this study, as those (48.9%) who are allocated in different regions than the capital (Lima) tend to fail their control appointments due to long traveling time/distance and self-financed expenses [24]. Therefore, the advantage of single-shot e-BT might be of special benefit for this group of subjects. The particular case of the Peruvian public healthcare system was herein presented; however, additional analysis in order to clarify the actual economic benefit of this approach in comparison to historical treatments and other latitudes is required.

## 4. Materials and Methods

### 4.1. Collective

**Figure 3:**
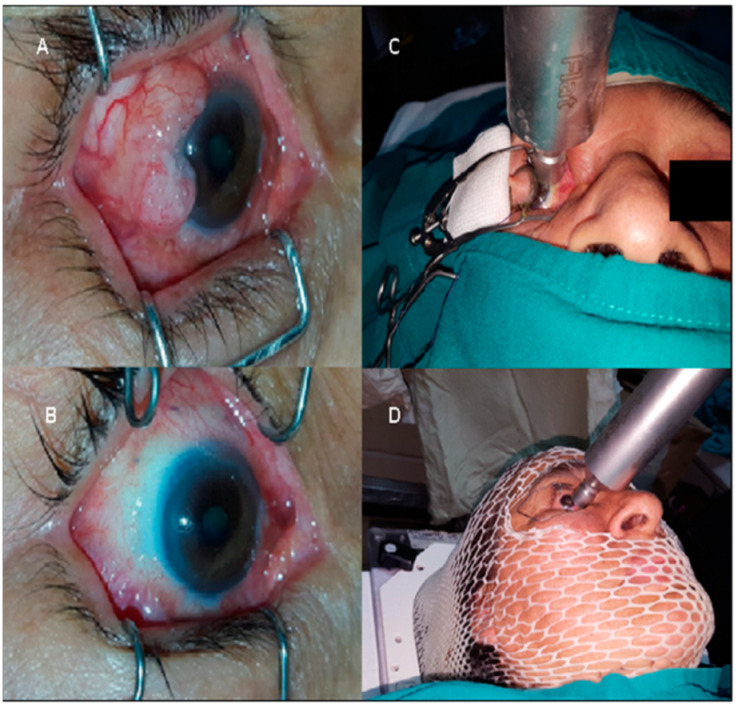
(**A**,**B**): Pre-surgery and post-e-BT status; (**C**,**D**): application procedure.

Patients diagnosed with primary T1–T3 infiltrating SCCC (AJCC TNM 8th edition) [56] of the limbal conjunctiva, who underwent surgery between October 2014 and January 2018 at Instituto Nacional de Enfermedades Neoplasicas, Lima, Peru (National Cancer Institute) and Instituto Nacional de Oftalmologia (National Ophthalmology Institute) with subsequently e-BT, were retrospectively assessed. All patients underwent wide local excision plus superficial keratectomy with 3–4 mm macroscopic margins, when feasible. Absolute alcohol was afterwards applied during ~30 s for all patients to the corneal margin. No cryotherapy was available at the institution. Adjuvant surface e-BT with a porTable 50 kV miniaturized X-ray source (Intrabeam, Carl Zeiss Meditec, Oberkochen, Germany) in a single fraction after definitive pathology report was performed (Figure 3). All histopathology reports were centralized and reviewed at our institution. Depending on margin involvement, e-BT doses ranged between 18 Gy for negative margins (R0) and 20–22 Gy for positive margin (R1) resections (according to the physician’s discretion and/or proximity to healthy structures). Depth prescription was normalized at 2 mm for a 90% isodose through a flat applicator, resembling equivalent doses in 2 Gy (EQD2) per fraction of 46–66 Gy (assuming α/β ratios of 8–10 Gy and a relative biological effectiveness [RBE] value of 1.3) [21,57] and yielding irradiation times between 5 to 7 min. The applicator’s diameter was selected according to the surgical bed diameter, under direct view and surgeon’s guidance during the procedure. Local anesthesia was applied before irradiation. Patients were required to fix the sight on the opposite direction of the application in order to avoid the lenses. For non-compliant patients, muscle blockade was indicated. For patients receiving treatment with a 2-cm applicator, an additional flat shield was placed over exceeding healthy tissue. An exemplary dose distribution simulation was retrospectively performed through a Monte Carlo-based calculation algorithm software (Radiance, GMV, Madrid, Spain), as the system was not available at the time of procedures. Post e-BT follow-up periods were performed every 3 months during the first year, 6 months until the second year, and annually afterwards. Complete ophthalmologic assessment in each visit and imaging for evaluating regional lymphatic stations (50 MhZ ultrasonography or CT-scan) every 6 months were performed.

Factors of interest included DFS and estimated rates, incidence of adverse events (graded according to the CTCAE v.4.03) [52] and toxicity-associated factors.

### 4.2. Statistical Analysis

A descriptive evaluation of the collected information, through frequencies and medians, was performed. Differences between patients groups with or without toxicity, regarding the quantitative characteristics, were assessed as per the *T* test for independent variables or its corresponding non-parametric test. The association between qualitative characteristics and toxicity was analyzed according to the Chi-square test, applying the Yates correction when needed. The DFS rate estimation was settled from the e-BT application date until the last follow-up, death, or locoregional recurrence date. Survival curves were estimated after the Kaplan–Meier method. A *p* < 0.05 value was established to define significance for associations or differences amongst variables. The statistical analysis was performed with the SPSS 22.0.0 software.

## 5. Conclusions

Electronic brachytherapy is a safe and effective tool for SCCC treatment in the short follow-up, with potential clinical and logistic advantages compared to classical EBRT or brachytherapy methods and chemotherapeutic agents. Longer follow-up and prospective assessment to confirm these findings are warranted.

## Figures and Tables

**Table 1:** Toxicity-related factors.

	Toxicity Profiles (%)		
	**G0**	**G2**	
	***n* = 35**	***n* = 12**	***p***
Age, years			
Median (Range)	69 (29–87)	72 (37–85)	0.855
T			
T1	15 (42.9)	4 (33.3)	1.000
T2	20 (57.1)	7 (58.3)	^†^ 1.000
T3	0 (0.0)	1 (8.3)	
Size, cm			
Mean (Range)	6 (2–20)	8.5 (1.5–20)	0.345
Weeks until application			
Median (Range)	10 (0–37)	8 (4–11)	0.354
Doses, Gy			
18	22 (62.9)	4 (33.3)	1.000
20	6 (17.1)	3 (25.0)	^†^ 1.000
22	7 (20.0)	5 (41.7)	
Applicator size			
1 cm	32 (91.4)	11 (91.7)	^†^ 1.000
2 cm	3 (8.6)	1 (8.3)	

Statistical assessment of toxicity-related factors. ^†^ Grouped categories. G0: No toxicity. G2: Topical intervention indicated.

**Table 2:** Exemplary dose-distribution profile of orbital risk structures.

	Doses Profile	
Structure	Max. Dose (Gy)	Volume (mL)
Lens	1.82	0.33
Optic nerve	0.14	0.69
Retina	5.52	2.75
Lacrimal gland	0.27	2.63

## Data Availability

Data not available.
